# Bacterial outer membrane vesicles at the plant–pathogen interface

**DOI:** 10.1371/journal.ppat.1006306

**Published:** 2017-06-01

**Authors:** Leron Katsir, Ofir Bahar

**Affiliations:** Department of Plant Pathology and Weed Research, Agricultural Research Organization, Volcani Center, Rishon LeZion, Israel; THE SAINSBURY LABORATORY, UNITED KINGDOM

## Overview

Gram-negative bacteria outer membrane vesicles (OMVs) are extracellularly released blebs, constantly detaching from the bacterial cell surface. Being ubiquitous among bacteria and diverse in content, OMVs have a plethora of functions: promoting virulence, mediating bacterial cell–cell communication, modulating host immune response, and more. Though most research on OMVs has been carried out on animal pathogens, production of OMVs by plant pathogenic bacteria is predicted to be similarly intrinsic to their biology. Recent studies in the field of plant–bacteria interactions have begun to unravel the roles of OMVs, showing their involvement in biofilm formation, virulence, and modulation of plant immunity. With a range of general to highly specialized roles, these structures can act as an adaptive toolbox during pathogenesis and stress. This Pearl will crystallize current OMV research with a special focus on the role OMVs play in plant–bacteria interactions.

## OMVs are intrinsic to Gram-negative bacteria

OMVs are formed continuously during growth and host colonization and are natural extensions of the bacteria producing them [1]. The phospholipid membrane bilayer of OMVs also contains lipopolysaccharides (LPS) and outer membrane–localized proteins. The OMV lumen envelops periplasmic constituents such as peptidoglycans (PG), soluble proteins, and enzymes and can contain an array of other small molecules, including RNA and DNA.

Released OMVs have been connected to several crucial bacterial behaviors, such as stress response, formation of biofilms, horizontal gene transfer, virulence, and cell–cell communication, and represent a general mechanism for the removal of misfolded toxic proteins [2][3]. As such, it is likely that OMV biogenesis and release are indispensable for bacteria and, thus far, mutants that do not produce OMVs have not been identified. The rate of vesicle production and the protein content of secreted OMVs vary when bacteria are grown under different environmental conditions, hinting at the existence of regulated biogenesis and cargo-sorting processes that direct specific proteins into OMVs [2,4]. The determinants that induce vesicle budding, the machinery that guides this process, and the rules governing incorporation or exclusion of specific proteins into OMVs are not clearly defined and are an active area of investigation [3][5][6][7][8][9]. The detection of virulence factors in OMVs from a wide range of bacteria, including plant pathogens, supports a general role for OMVs in promoting pathogenesis [10][11]. Resolving the distinction between proteins secreted selectively for pathogenesis against those selectively removed for bacterial health or simply due to abundance will be a key challenge in interpreting the proteomic data being generated for diverse bacterial OMVs [6]. The fact that OMVs are naturally and regularly produced with a broad range of constituents, representing a multifunctional secretion pathway, sets the stage for inevitable multifarious interactions with the host environment.

## OMVs are carriers of multiple immune elicitors and modulate plant immunity

**Fig 1:**
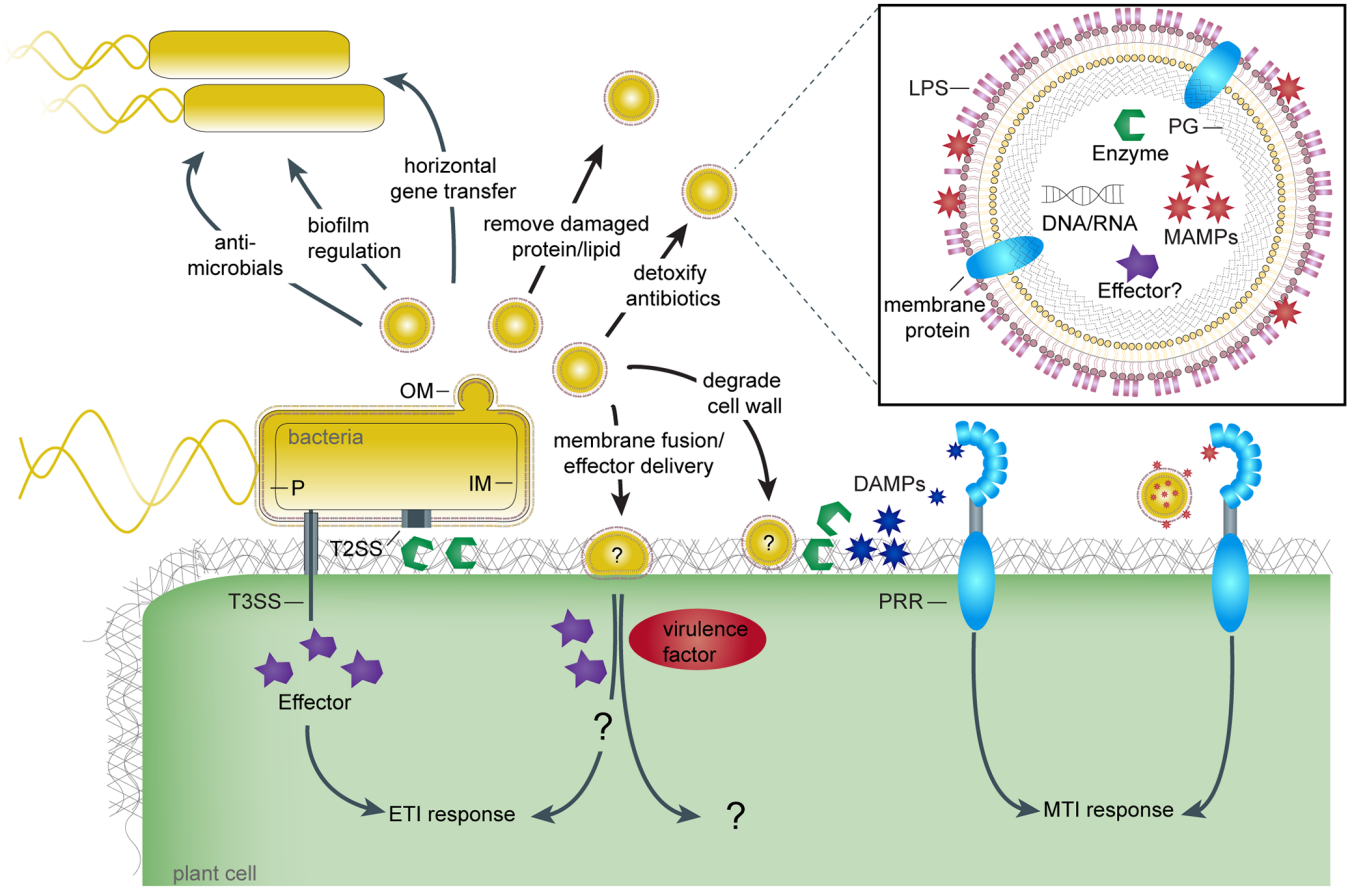
The myriad functions of bacterial outer membrane vesicles. This figure illustrates the blebbing of outer membrane vesicles (OMVs, spherical yellow structures) from the bacterial cell (yellow rod shape) and the processes they participate in or potentially participate in. Functions related to bacterial cell–cell interactions, such as regulation of biofilm, horizontal gene transfer, removal of damaged molecules, and so on, have been demonstrated in several bacterial species [2][3]. OMVs have been shown to activate the plant immune system [14]. A close-up illustration of an OMV (upper right) shows it contains various known microbe-associated molecular patterns (MAMPs) such as lipopolysaccharides (LPS), peptidoglycans (PG), and proteinaceous MAMPs that can potentially interact with pattern recognition receptors (PRRs) and induce MAMP-triggered immunity (MTI). Other relevant features of OMVs are the release of plant cell wall–degrading enzymes, which may assist in bacterial virulence [21][23] but may also induce MTI via the generation of damage-associated molecular patterns (DAMPs). OMVs can fuse to target cells, be it a bacterial or a mammalian host cell [16][18]; here we illustrate the hypothetical fusion of an OMV with a plant cell. Since OMVs have been shown to carry type III secreted (T3S) effectors, these could potentially be introduced into the host cells by OMV delivery and either promote host susceptibility or resistance by activating effector-triggered immunity (ETI), though this has yet to be demonstrated. OMVs may carry other virulence factors that could be introduced into the host to promote pathogenesis in yet uncharacterized molecular mechanisms. OM, outer membrane; IM, inner membrane; P, periplasm; T2SS, Type II secretion system; T3SS, Type III secretion system.

Pathogen recognition is essential for the host to mount an effective immune response. Host cells may monitor for OMVs as a cue for pathogen invasion by recognizing OMV microbe-associated molecular patterns (MAMPs) [12]. The perception of OMVs in mammalian systems is facilitated by cell surface and cytosolic receptor recognition of OMV MAMPs and was recently reviewed [13]. Plants have only recently been shown to recognize and respond to OMVs purified from plant pathogens by activating typical innate immune responses [14]. MAMP diversity in OMVs is large and ranges from integral elements like LPS and PG to variable proteinaceous cargo such as Elongation Factor-Tu (EF-Tu) and flagellin, which have been found to be associated with purified OMVs [3,4,10,13]. This complex array of immune elicitors can be recognized by plant immune receptors known as pattern recognition receptors (PRRs), which have extracellular domains for MAMP recognition (Fig 1) [15].

Mutants of the model plant *Arabidopsis* lacking single PRRs show little or no change in the induction of defense responses following OMV treatment. This observation, together with biochemical evidence for the association of known MAMPs with OMVs, may suggest that OMVs can activate multiple PRRs, reflecting the redundancy of immune receptor signaling pathways potentially activated by OMVs [14]. Furthermore, an *Arabidopsis* mutant line producing a nonfunctional version of the co-receptor brassinosteroid-insensitive 1–associated kinase (BAK1), which is known to be a signalling hub for multiple PRRs, showed a significantly reduced response to OMVs, strengthening the hypothesis that multiple PRRs are responsive to and activated by OMVs [14]. A similar response, albeit slightly less significant, was seen with an *Arabidopsis* knockout line of the co-receptor Suppressor of BIR1-1 (SOBIR1) [14]. Intriguingly, EF-Tu was found to be associated with OMVs and to be recognized by its respective plant immune receptor [14]. The fact that EF-Tu is associated with OMVs from a broad range of bacteria suggests that OMVs could represent a conserved secretion pathway for this protein [5]. Whether bacterial OMVs can enter the plant cell or whether there are plant cytosolic receptors to detect them is unknown. However, clathrin-mediated endocytosis of OMVs is an element of the mammalian immune surveillance system for pathogens like enterohemorrhagic *Escherichia coli*, for which internalization of OMVs allows cytosolic sensing of LPS [16]. Does the presence of the plant cell wall prevent OMV fusion to the plant cell plasma membrane? Future studies should shed light on this. Moreover, what is the outcome of these interactions, and how do OMV-induced immune responses differ from or contribute to those caused directly by the bacterium? The lack of bacterial mutants that do not produce OMVs makes resolving this problem and clarifying the role of OMVs from that of their constituents produced by bacteria even more challenging. These are just some of the questions that will probably challenge the field of plant–microbe interactions in the future.

## The OMV secretion pathway facilitates the bacterial infection process

OMVs are beneficial to bacterial pathogens in the context of host colonization [3]. In addition to mitigating the effects of host-produced antibiotics through increased vesiculation, OMVs can shield the bacterial body from antibiotics by carrying enzymes that mediate antibiotic protection [17]. The packaging and delivery of key molecules and enzymes in and by OMVs have implications in regulating host development, biofilm formation, nutrient acquisition, and in promoting disease (Fig 1).

One of the unique features of the OMV secretory pathway is the ability to coordinately deliver multiple effector molecules simultaneously to the target site. One example of this capability is provided by *Pseudomonas aeruginosa*, which releases OMVs loaded with virulence factors, protectively traveling to the target site at the host cytoplasmic membrane, where the vesicle fuses with the host membrane to deliver the cargo into the cytoplasm [18]. This phenomenon resembles, in a way, the effective and directed delivery of type III-secreted (T3S) effectors by certain plant pathogens into the host cytoplasm (Fig 1, [19]). OMVs, in contrast to the T3S system, have the potential to deliver a much larger and diverse array of molecules to the host cytoplasm without the requirement of direct proximity between the bacteria and host cell.

Although OMV fusion with plant cells is yet to be shown, OMVs do contribute to bacterial virulence *in planta*. In the xylem-inhabiting plant pathogen *Xylella fastidiosa*, quorum sensing through the production and perception of diffusible signaling factor (DSF) induces aggregation, surface attachment, and biofilm formation. In a recent report by Ionesco et al. (2014), it was shown that a lack of DSF production, as in the Δ*rpfF* mutant, promotes a more virulent-free swimming form of the bacteria that overproduces OMVs. The authors suggest that OMVs serve as anti-adherence factors participating in mediating the switch from a sessile biofilm form of the bacterium to a free-swimming form, facilitating cell dispersion in the xylem and promoting virulence [20].

The OMVs secretory pathway has also been found to serve as an alternative route for extracellular enzymes secreted by the type II secretion (T2S) system [21]. Enzymes such as lipases, proteases, and cell wall–modifying proteins found in proteomics analyses of OMVs [10][22] could be the source of damage-associated molecular patterns (DAMPs) that are also recognized by PRRs. In fact, there seems to be an overlap in the enzymes secreted via the T2S system and those packaged into OMVs. T2S xylanase, a plant cell wall–degrading enzyme whose secretion into the extracellular space is important for *Xanthomonas campestris* virulence, was shown to be secreted by both the T2S system and by OMVs [21]. Other virulence factors take the same route, as well. For instance, the packaging of LesA, a lipase/esterase also secreted by the T2S system in *X*. *fastidiosa*, into OMVs promotes the spread of disease symptoms [23]. This indicates that OMVs are an alternative route for T2S substrates and important for plant pathogen disease progression [21][23]. The packaging of LesA and xylanase into OMVs has several advantages over the T2S route: it can broaden the effective range of activity by protecting the protein from the extracellular environment and can allow targeted and coordinated delivery simultaneously [24]. The detection of T3S effector proteins and proteins related to their transport in plant pathogen OMVs suggests that OMVs could also be an alternative pathway for the T3S system or act in coordination with it [22][10].

OMVs could thus facilitate delivery of virulence factors both proximal and distal to the site of bacteria. Understanding the mechanisms of delivery as well as how OMV production cooperates with the other bacterial secretion systems will clarify how bacteria influence their extracellular environments.

## Future perspective

Clearly, understanding the biology of plant–bacteria interactions is not complete without accounting for OMVs. The multitude of roles played by these extracellular organelles, from immune modulation to regulation of biofilms, nutrient acquisition, protein secretion, and detoxification, makes them a multifunction tool, much like a Swiss Army knife, available to respond to a variety of challenges. Whether bacteria can in fact select the tool set or if the OMV is only a microcosm of what is synthesized in the bacterial body remains to be seen. The rich species diversity of plant pathogenic bacteria offers many avenues for investigation into the way bacteria utilize these tools in specific plant–bacteria interactions. Beyond plant pathogenic bacteria, other microorganisms such as plant pathogenic fungi, nematodes, and also mutualistic microorganisms like rhizobia are likely to secrete extracellular vesicles [25,26]. The role of these extracellular vesicles in pathogenesis and in symbiosis is a fascinating area of research. It is clear we have barely crossed the outer membrane of plant–bacteria OMV research.

## References

[1] Kulp A, Kuehn MJ. Biological functions and biogenesis of secreted bacterial outer membrane vesicles. Annu Rev Microbiol. 2010;64: 163–184. 10.1146/annurev.micro.091208.073413 20825345 PMC3525469

[2] McBroom AJ, Kuehn MJ. Release of outer membrane vesicles by Gram-negative bacteria is a novel envelope stress response. Mol Microbiol. 2007;63: 545–558. 10.1111/j.1365-2958.2006.05522.x 17163978 PMC1868505

[3] Schwechheimer C, Kuehn MJ. Outer-membrane vesicles from Gram-negative bacteria: biogenesis and functions. Nat Rev Microbiol. 2015;13: 605–19. 10.1038/nrmicro3525 26373371 PMC5308417

[4] Altindis E, Fu Y, Mekalanos JJ. Proteomic analysis of Vibrio cholerae outer membrane vesicles. Proc Natl Acad Sci U S A. 2014;111: E1548–56. 10.1073/pnas.1403683111 24706774 PMC3992640

[5] Lee J, Kim OY, Gho YS. Proteomic profiling of Gram-negative bacterial outer membrane vesicles: Current perspectives. Proteomics Clin Appl. 2016; 1–41. 27480505 10.1002/prca.201600032

[6] Cahill BK, Seeley KW, Gutel D, Ellis TN. Klebsiella pneumoniae O antigen loss alters the outer membrane protein composition and the selective packaging of proteins into secreted outer membrane vesicles. Microbiol Res. 2015;180: 1–10. 10.1016/j.micres.2015.06.012 26505306

[7] Schertzer J, Whiteley M. A Bilayer-Couple Model of Bacterial Outer Membrane Vesicle. MBio. 2012;3: e00297–11. 10.1128/mBio.00297-11 22415005 PMC3312216

[8] Schwechheimer C, Sullivan CJ, Kuehn MJ. Envelope control of outer membrane vesicle production in Gram-negative bacteria. Biochemistry. 2013;52: 3031–3040. 10.1021/bi400164t 23521754 PMC3731998

[9] Kadurugamuwa JL, Beveridge TJ. Virulence factors are released from Pseudomonas aeruginosa in association with membrane vesicles during normal growth and exposure to gentamicin: A novel mechanism of enzyme secretion. J Bacteriol. 1995;177: 3998–4008. 7608073 10.1128/jb.177.14.3998-4008.1995 PMC177130

[10] Sidhu VK, Vorhölter F-J, Niehaus K, Watt SA. Analysis of outer membrane vesicle associated proteins isolated from the plant pathogenic bacterium Xanthomonas campestris pv. campestris. BMC Microbiol. 2008;8: 87 10.1186/1471-2180-8-87 18518965 PMC2438364

[11] Kuehn MJ, Kesty NC. Bacterial outer membrane vesicles and the host—pathogen interaction. Genes Dev. 2005; 2645–2655. 10.1101/gad.1299905 16291643

[12] Ellis TN, Leiman SA, Kuehn MJ. Naturally produced outer membrane vesicles from Pseudomonas aeruginosa elicit a potent innate immune response via combined sensing of both lipopolysaccharide and protein components. Infect Immun. 2010;78: 3822–3831. 10.1128/IAI.00433-10 20605984 PMC2937433

[13] Kaparakis-Liaskos M, Ferrero RL. Immune modulation by bacterial outer membrane vesicles. Nat Rev Immunol. 2015;15: 375–387. 10.1038/nri3837 25976515

[14] Bahar O, Mordukhovich G, Luu DD, Schwessinger B, Daudi A, Jehle AK, et al Bacterial Outer Membrane Vesicles Induce Plant Immune Responses. Mol Plant Microbe Interact. 2016;29: 374–384. 10.1094/MPMI-12-15-0270-R 26926999

[15] Trdá L, Boutrot F, Claverie J, Brulé D, Dorey S, Poinssot B. Perception of pathogenic or beneficial bacteria and their evasion of host immunity: pattern recognition receptors in the frontline. Front Plant Sci. 2015;6: 219 10.3389/fpls.2015.00219 25904927 PMC4389352

[16] Vanaja SK, Russo AJ, Behl B, Banerjee I, Yankova M, Deshmukh SD, et al Bacterial Outer Membrane Vesicles Mediate Cytosolic Localization of LPS and Caspase-11 Activation. Cell. 2016;165: 1106–1119. 10.1016/j.cell.2016.04.015 27156449 PMC4874922

[17] Lee J, Lee EY, Kim SH, Kim DK, Park KS, Kim KP, et al Staphylococcus aureus extracellular vesicles carry biologically active -lactamase. Antimicrob Agents Chemother. 2013;57: 2589–2595. 10.1128/AAC.00522-12 23529736 PMC3716153

[18] Bomberger JM, Maceachran DP, Coutermarsh B a, Ye S, O’Toole G a, Stanton B a. Long-distance delivery of bacterial virulence factors by Pseudomonas aeruginosa outer membrane vesicles. PLoS Pathog. 2009;5: e1000382 10.1371/journal.ppat.1000382 19360133 PMC2661024

[19] Galan JE, Collmer A. Type III Secretion Machines: Bacterial Devices for Protein Delivery into Host Cells. Science. 1999;284: 1322–1329. 10.1126/science.284.5418.1322 10334981

[20] Ionescu M, Zaini PA, Baccari C, Tran S, da Silva AM, Lindow SE. Xylella fastidiosa outer membrane vesicles modulate plant colonization by blocking attachment to surfaces. Proc Natl Acad Sci. 2014;111: E3910–E3918. 10.1073/pnas.1414944111 25197068 PMC4169949

[21] Solé M, Scheibner F, Hoffmeister A-K, Hartmann N, Hause G, Rother A, et al Xanthomonas campestris pv. vesicatoria secretes proteases and xylanases via the Xps-type II secretion system and outer membrane vesicles. J Bacteriol. 2015;197: 2879–2893. 10.1128/JB.00322-15 26124239 PMC4524030

[22] Chowdhury C, Jagannadham MV. Virulence factors are released in association with outer membrane vesicles of Pseudomonas syringae pv. tomato T1 during normal growth. Biochim Biophys Acta—Proteins Proteomics. 2013;1834: 231–239. 10.1016/j.bbapap.2012.09.015 23043909

[23] Nascimento R, Gouran H, Chakraborty S, Gillespie HW, Almeida-Souza HO, Tu A, et al The Type II Secreted Lipase/Esterase LesA is a Key Virulence Factor Required for Xylella fastidiosa Pathogenesis in Grapevines. Sci Rep. 2016;6: 18598 10.1038/srep18598 26753904 PMC4709584

[24] Galka F, Wai SN, Kusch H, Engelmann S, Hecker M, Schmeck B, et al Proteomic characterization of the whole secretome of Legionella pneumophila and functional analysis of outer membrane vesicles. Infect Immun. 2008;76: 1825–1836. 10.1128/IAI.01396-07 18250176 PMC2346698

[25] Quintana JF, Babayan SA, Buck AH. Small RNAs and extracellular vesicles in filarial nematodes: from nematode development to diagnostics. Parasite Immunol. 2016; 10.1111/pim.12395 27748953

[26] Peres da Silva R, Puccia R, Rodrigues ML, Oliveira DL, Joffe LS, César G V, et al Extracellular vesicle-mediated export of fungal RNA. Sci Rep. 2015;5: 7763 10.1038/srep07763 25586039 PMC5379013

